# The Multiradical Character of One- and Two-Dimensional Graphene Nanoribbons**

**DOI:** 10.1002/anie.201207671

**Published:** 2013-01-28

**Authors:** Felix Plasser, Hasan Pašalić, Martin H Gerzabek, Florian Libisch, Rafael Reiter, Joachim Burgdörfer, Thomas Müller, Ron Shepard, Hans Lischka

**Affiliations:** Institute for Theoretical Chemistry, University of ViennaWaehringerstrasse 17, 1090 Vienna (Austria); Institute of Soil Research, University of Natural Resources and Life SciencesPeter-Jordan-Strasse 82, 1190 Vienna (Austria); Institute for Theoretical Physics, Vienna University of TechnologyWiedner Hauptstrasse 8–10, 1040 Vienna (Austria); Institute of Advanced Simulation, Jülich Supercomputer Centre, Forschungszentrum Jülich52425 Jülich (Germany); Chemical Sciences and Engineering Division, Argonne National LaboratoryArgonne, IL 60439 (USA); Department of Chemistry and Biochemistry, Texas Tech UniversityLubbock, TX 79409-1061 (USA)

**Keywords:** chemical stability, graphene, multiradical character, multireference methods, quantum chemistry

Since the first experimental realizations of graphene nanoribbons,^1^ graphene nanodevices^2^ have attracted enormous attention in the quest for future nanoscale technologies. Because of their small band gaps and high charge-carrier mobilities, *n*-acenes (Figure 1) and functionalized acenes are being considered as highly interesting building units for organic electronic materials.^3^ Zigzag nanoribbons feature remarkable spin-polarization and half-metallic properties^4^ and chemically fascinating challenges have to be solved in the synthesis of such extended polyaromatic hydrocarbons (PAHs).^5^ Moreover, the chemical properties of graphene nanoribbons may be linked to the reactivity of black carbon surfaces and PAHs in soils, affecting the accumulation of persistent organic pollutants with environmental implications.^6^

**Figure 1:**
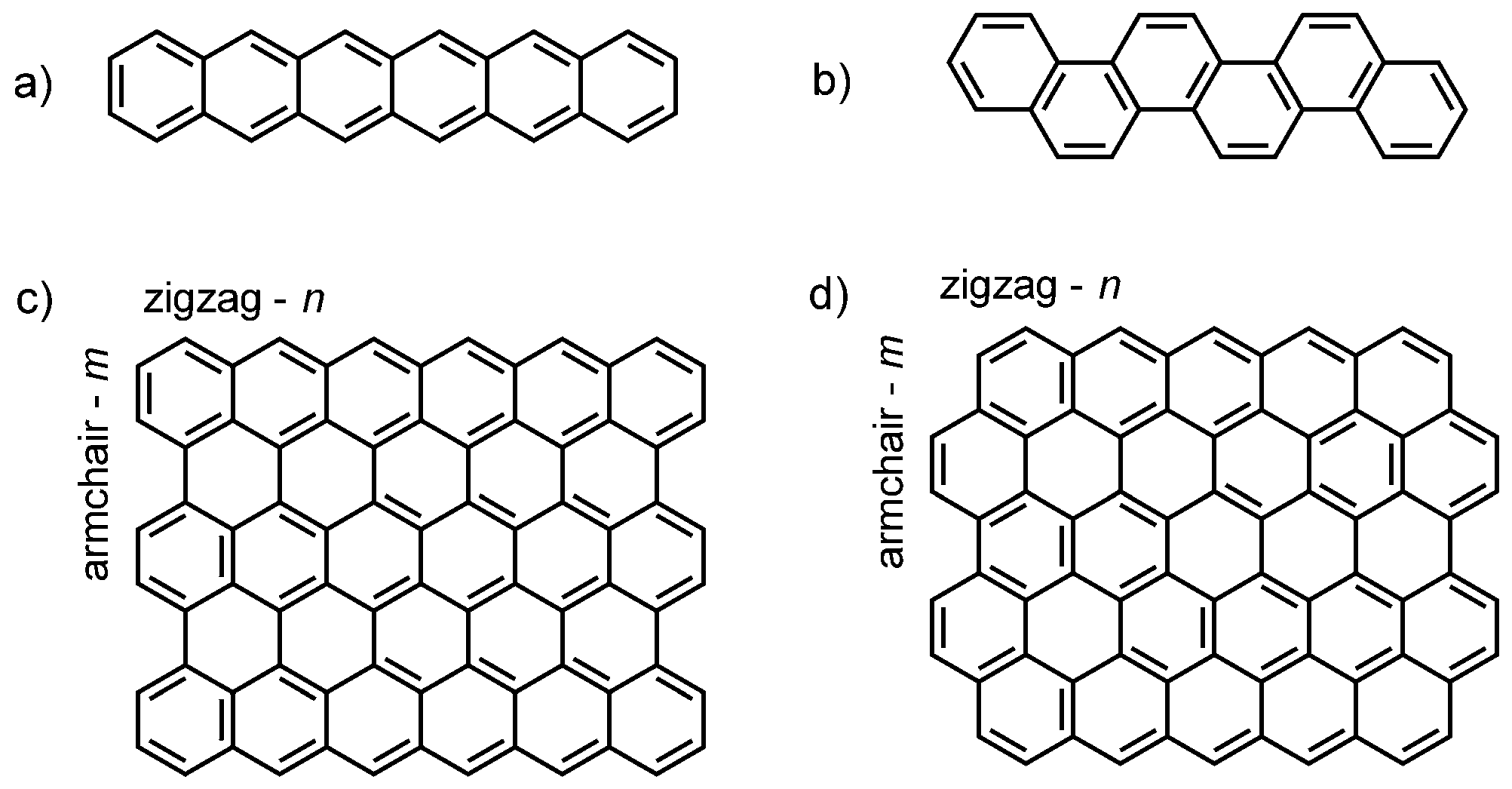
Structures investigated: a) *n*-acene, b) *n*-phenacene, c) (*m*a, *n*z) periacene, and d) (*m*a, *n*z) circumacene.

The availability of longer acenes is, however, hampered by their increasing reactivity, with pentacene being the largest well-characterized acene.3b In recent years substantial progress has resulted in the synthesis of *n*-acenes up to *n*=9 by matrix isolation techniques (see Ref. 7 and references therein). Nevertheless, these higher acenes are very reactive; for example, heptacene was found to be stable only for 4 h in a poly(methyl methacrylate) matrix.^8^ To overcome the stability problems, larger acenes were functionalized7a by adding protecting groups which inhibit the native high reactivity of the acenes.

Quantum chemical investigations play a major role in clarifying the outstanding electronic properties of carbon nanoflakes displaying bi- or even multiradical character. Density functional theory (DFT) constitutes a natural choice because of its good performance in terms of accuracy and computational efficiency with numerous applications to acenes^9^ and graphene flakes.^10^ However, because of the pronounced radical character, unrestricted DFT methods must be used,9a which are afflicted with spin contamination problems and the challenge of choosing a proper functional. As alternatives, density matrix renormalization group (DMRG)^11^ and the active-space variational two-electron reduced-density-matrix (2-RDM) approaches have been applied to one-12 and some two-dimensional^13^ systems. Additionally, spin-flip configuration interaction^14^ coupled cluster with singles and doubles (CCSD), along with more extended calculations, have been performed^15^ to investigate the electronic structure of the *n*-acenes.

For an adequate treatment of multiradical systems multireference (MR) methods^16^ are especially well suited. Moreover, MR methods are not affected by spin contamination or energy instability problems. In the present work we employ the MR averaged quadratic coupled cluster method (MR-AQCC)^17^ which includes crucial size-extensivity corrections at the MR level. It has already been successfully applied to several smaller challenging biradical systems.^18^ We show here its applicability to significantly larger molecular systems in the range of 100 carbon atoms in the graphene nanoflake.

The analysis of the radical character of PAHs as model systems for graphene nanoflakes will be performed by considering the natural orbital (NO) occupations as computed from the AQCC density by 1) following the deviations of individual NO occupations *n_i_* from zero (unoccupied) and two (doubly occupied), respectively, and by 2) computing a density and a number of effectively unpaired electrons *N_U_* as originally introduced by Takatsuka et al.^19^ as the distribution of “odd” electrons, which provides a measure for the splitting of an electron pair into different spatial regions (see also Ref. 20). We use the formalism of Ref. 21 and the nonlinear formula *n_i_*^2^(2−*n_i_*)^2^ given in this reference to compute the density of unpaired electrons; it more cleanly separates the dynamical correlation contributions to the wave function from the truly open-shell contributions of the radical centers. Specifically, relative to the linear function also suggested in Ref. 21, it emphasizes contributions from orbitals with occupations near 1, while also suppressing contributions with occupations near 0 or 2.

Two classes of molecular systems (Figure 1) have been investigated: 1) quasi-one-dimensional *n*-acenes and *n*-phenacenes and 2) two-dimensional periacenes and circumacenes. The latter will be characterized by the notation (*m*a, *n*z) where *m* and *n* count the number of benzene rings along each direction and a and z denote armchair and zigzag boundaries, respectively (see Figure 1 c,d). The latter two structures differ only in the details of their armchair edges leading, however, to significant changes in the radical character.

In our approach we follow the strategy of the DMRG^11^ and 2-RDM^12^ calculations in focusing on the conjugated π system. This is achieved by freezing the σ orbitals at the self-consistent field (SCF) level. The molecular orbitals (MOs) are obtained from multiconfiguration (MC) SCF calculations for use in the subsequent MR-AQCC approach. In all cases the totally symmetric singlet state is computed. The active orbital space for the reference configurations was chosen such that polyradical character and several open-shell electrons could be properly represented. Two reference spaces including up to 16 active orbitals have been selected for the MR-AQCC calculations to account for the exceptional requirements of the different compounds. The basis set used is of double-zeta quality. More details on the computational methods can be found in the Supporting Information.

The acene series (Figure 2) exhibits a dramatic increase of the open-shell NO occupations with chain length *n*, resulting in an entire cascade of open-shell occupations. For *n*=11 two NOs are almost degenerate (with occupations of 1.1 *e* and 0.90 *e*), but even the neighboring orbital occupations (1.6 *e* and 0.4 *e*) easily qualify as open-shell orbitals. The increasing instability of the acenes with chain length correlates well with the increasing multiradical character displayed in Figure 2. On the other hand, the NO occupations for the phenacenes (Figure S1 in the Supporting Information) do not show any indication of biradical character up to *n*=10, a chain length at which the acenes already possess strong multiradical character.

**Figure 2:**
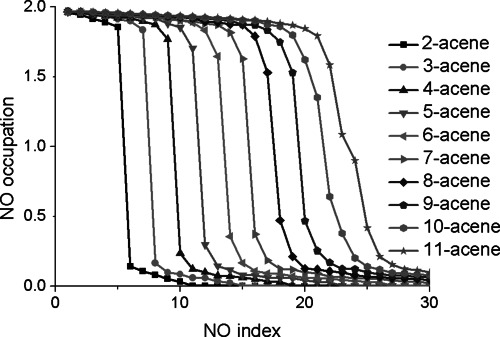
Evolution of MR-AQCC NO occupations with the length *n* of the acene chain.

The evolution of strong multiradical character in our calculations agrees well with the DMRG^11^ and 2-RDM^12^ results, but is in contrast to the recent CCSD calculations^15^ where from the analysis of the T1 diagnostics it was concluded that the acenes are of pure closed-shell character. However, it has been discussed intensively in the literature^22^ that the single excitations involved in this diagnostic are associated with orbital relaxation and that the double excitations must be analyzed to assess the multireference character of a CCSD calculation. CCSD calculations using the D2 diagnostic developed for that purpose22b have been performed^23^ using the PSI4 program system.^20^ The D2 diagnostic (see Table S2 in the Supporting Information) considerably exceeds the recommended threshold; therefore, by this measure, the CCSD method also displays significant multireference character, in good agreement with our MR-AQCC calculations.

Extending this analysis from quasi one-dimensional quantum wires to true two-dimensional nanoflakes, we investigate both (3a, *n*z) and (5a, *n*z) periacenes (Figure S2 and Figure 3) and the circumacene sequences (Figure S3 and Figure 5). Similar to the quasi-1D acenes, the two-dimensional periacene sequence develops a strong multiradical character with increasing zigzag chain length. This evolution is more rapid than in the acene case. Increasing the armchair length in the periacenes from *m*=3 to 5 (compare Figure S2 and Figure 3) enhances the open-shell character considerably.

**Figure 3:**
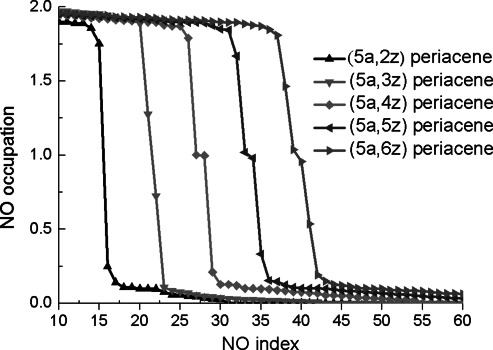
Dependence of MR-AQCC NO occupations on the zigzag length in (5a, *n*z) periacenes.

For the 5a series (Figure 3) complete degeneracy in the NO occupations is found already for *n*=4. The radical character is predominantly located at the zigzag edges as shown for the (5a, 6z) case (Figure 4). In this case the number of effectively unpaired electrons *N_U_* is 5.0 *e*. Individual values attributed to the carbon atoms based on a Mulliken analysis^24^ of the density of unpaired electronsare given in Figure 4 as well. They illustrate the strong radical character and the related chemical reactivity at the zigzag edges. The four orbitals of strong open-shell character for (5a, 6z) periacene contain about three open-shell electrons. The difference to the *N_U_* value arises from the contribution of the remaining minor open-shell occupation included in the density of unpaired electrons. Spin-polarized DFT calculations on rectangular nanoribbons using the Heyd, Scuseria, and Ernzerhof (HSE06) hybrid density functional show10a that spin polarization sets in already at a relatively small size of (3a, 3z). The MR-AQCC NO occupations (Figure S2) lead to a similar finding concerning this onset but give, additionally, a quantitative picture of the extent of the radical generation along the periacene series.

**Figure 4:**
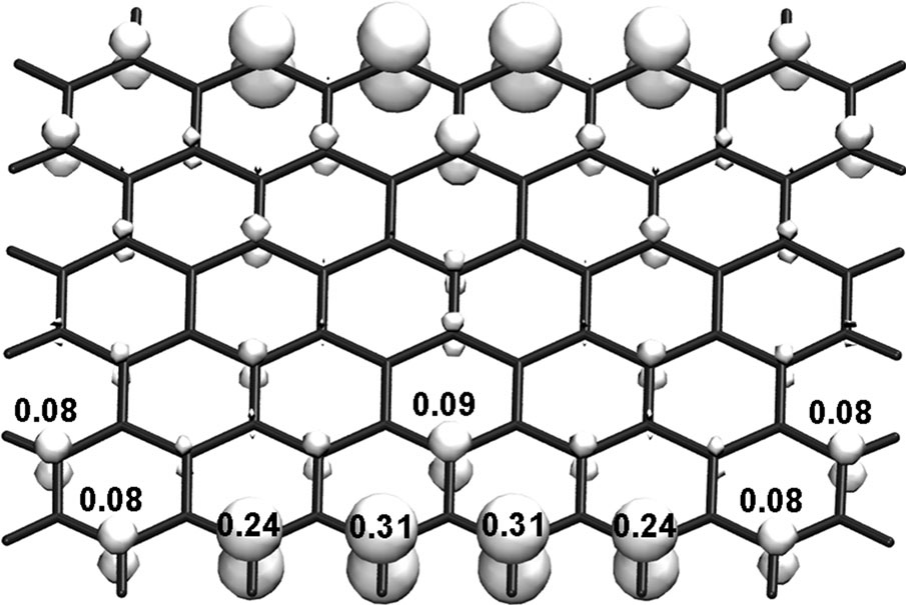
Density of unpaired electrons for (5a, 6z) periacene (isovalue 0.005 *e*). *N_U_*=5.0 *e*, individual atomic values are given next to the respective carbon atoms.

A notably delayed appearance of biradical character is found for the (3a, *n*z) and (5a, *n*z) circumacenes (Figure S3 and Figure 5, respectively) in comparison to the periacene pattern (Figure 3). In the latter case an NO pair with occupations of roughly 0.75 *e* and 1.25 *e* appears already for *n*=3, whereas in the (5a, *n*z) circumacene series comparable occupation values are obtained only for *n*=5. For higher members of the circumacene series eventually a similar cascade of open-shell occupations is found as for the periacenes.

**Figure 5:**
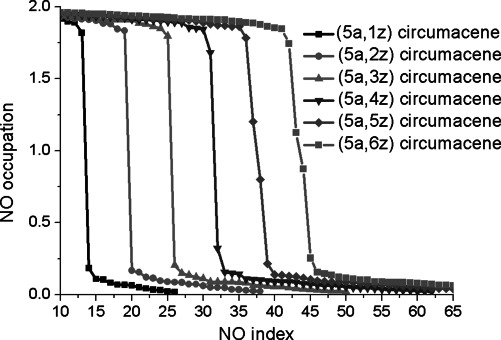
Dependence of NO occupations on zigzag length *n* in (5a, *n*z) circumacenes.

The density of unpaired electrons for the (5a, 6z) circumacene (Figure S4) closely resembles the one for the corresponding (5a, 6z) periacene. Consequently, we predict the circumacenes to approach with increasing length the radical character and reactivity of the periacenes. Jiang et al.10d have used the simple HOMO–LUMO gap criterion to estimate a critical value of *n_C_*=6 for (3a, *n*z) circumacenes to reach open-shell character. This result is in qualitative agreement with ours, even though the numbers of effectively unpaired electrons discussed below indicate the transition to significant open-shell character occurs already between *n*=4 and *n*=5.

According to the *N_U_* analysis (Figure 6) the graphene nanoflakes can be subdivided into three groups. The *n*-phenacenes show a practically horizontal line with *N_U_* values of approximately 0.3–0.5 *e*. They exemplify the closed-shell character within our analysis. Next, the *n*-acenes show the strong increase in unpaired character reaching an *N_U_* value of 4 *e* for *n*=11. This value is in good agreement with the four open-shell NOs shown in Figure 2. The third class of compounds is formed by the two-dimensional graphene flakes. They all show an increase in the unpaired electron character which is notably stronger than that of the acenes up to *n*=9. There are differences in the behavior of periacenes and circumacenes: the strong increase in *N_U_* with the chain length *n* starts immediately for the periacenes whereas in particular the (3a, *n*z) circumacenes start off more slowly.

**Figure 6:**
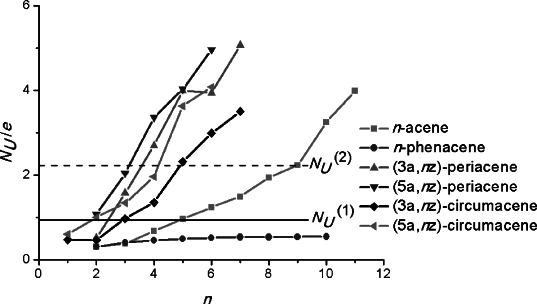
Dependence of the number of effectively unpaired electrons *N_U_* with chain length *n*.

It is now tempting to correlate the *N_U_* values of different classes of graphene nanoflakes with available experimental evidence for the reactivity. Accordingly, we can identify a region of stability below the critical line *N_U_*^(1)^≍1 *e* passing through pentacene, the largest well-accessible member in the acene series.3b This region includes the periacenes (3a, 2z) (perylene) and (5a, 2z), and the circumacenes (3a, 1z) (pyrene), (3a, 2z) (coronene, circumbenzene), (3a, 3z) (ovalene, circumnaphthalene), (5a, 1z) and (5a, 2z), all of which are known stable compounds.^25^ The synthesis of circumanthracene has also been reported.^26^ A somewhat more tentative assignment is the region delimited by the dashed line *N_U_*^(2)^=2.2 *e* passing through nonacene, the largest acene synthesized so far.7b It is interesting to note that the *N_U_* values for the acenes show an additional increase at *n*=9 indicating an even heightened difficulty in preparing acenes with *n*>9. This second region at or below *N_U_*^*(*2)^ would suggest that two-dimensional flakes including the (3a, 3z) and (5a, 3z) periacenes and circumacenes up to (5a, 4z) should be synthesizable under similar precautions as those used for the higher *n*-acenes. For larger 2D nanoflakes currently available,1b we conjecture that the different scaling of circumference versus area results in increased stability in the bulk region, as the density of unpaired electrons is restricted to the edges (Figure 4). The comparison of the properties of the different PAHs investigated in this work illustrates the remarkable variety in open-shell character when different pathways are followed in extending the molecular size of PAHs. The analysis of different PAH topologies in terms of structural and electronic properties is of considerable chemical interest.^27^ we have shown that MR methods provide powerful tools for obtaining reliable information on these fascinating compounds which are so difficult to access experimentally.

## Supplementary material

As a service to our authors and readers, this journal provides supporting information supplied by the authors. Such materials are peer reviewed and may be re-organized for online delivery, but are not copy-edited or typeset. Technical support issues arising from supporting information (other than missing files) should be addressed to the authors.
