# Supplementary material for: The Multiradical Character of One- and Two-Dimensional Graphene Nanoribbons
Source: Angew Chem Int Ed Engl. 2013 Jan 28;52(9):2581–4. doi: 10.1002/anie.201207671 (PMC3648980; doi:10.1002/anie.201207671)
Supplement: Supplementary file 1 [file anie0052-2581-SD1.pdf]

Angewandte Chemie
Eine Zeitschrift der Gesellschaft Deutscher Chemiker

Supporting Information

© Wiley-VCH 2013

69451 Weinheim, Germany

The Multiradical Character of One- and Two-Dimensional Graphene Nanoribbons**

*Felix Plasser, Hasan Pašalić, Martin H. Gerzabek, Florian Libisch, Rafael Reiter, Joachim Burgdörfer, Thomas Müller, Ron Shepard, and Hans Lischka**

anie_201207671_sm_miscellaneous_information.pdf

Table of Contents

Page

1. Computational Methods 1
2. Table S1: Orbital occupation specification for the RAS(6)/CAS(4,4)/AUX(6)-1ex used in MCSCF and MR-AQCC calculations on polyacenes with k condensed rings. 3
3. Table S2: D1 and D2 diagnostic values for the *n*-acene series using the CCSD method 4
4. Figure S1. Evolution of MR-AQCC NO occupations with the length of the phenacene chain. 5
5. Figure S2. Dependence of MR-AQCC NO occupations on the zigzag length in (3a,*n*z) periacenes. 6
6. Figure S3. Dependence of NO occupations on zigzag length *n* in (3a,*n*z) circumacenes. 7
7. Figure S4. Unpaired electron density for (5a,6z) circumacene (isovalue 0.005 *e*). The total number of unpaired electrons N_U is 4.1 *e*, individual atomic values are given next to the respective carbon atoms. 8
8. References 9

Computational Methods

Multiconfiguration self consistent field (MCSCF) calculations were performed to create the molecular orbitals (MOs) to be used in the subsequent multireference averaged quadratic coupled cluster (MR-AQCC) calculations. Two sets of active spaces have been used at MCSCF level denoted a) RAS(6)/CAS(4,4)/AUX(6)-1ex and b) CAS(8,8). RAS stands for restricted active space containing six orbitals which are initially doubly occupied, AUX for auxiliary space and CAS for complete active space. In active space a) the 16 active electrons were distributed over the remaining orbitals with the restrictions that at least 11 electrons were in RAS (single excitations), and at most one was located in AUX. The detailed orbital occupation is specified below. Active space a) was used for the acene calculations and space b) for the pericene and circumacene calculations. The CAS orbitals in space a) describe the open shell occupancies, and the RAS and AUX orbitals were chosen to include higher excitations at MR-AQCC level and to avoid intruder states at that level. The reference spaces for the MR-AQCC calculations were identical to the active spaces described for the MCSCF calculations. Using these references, all single and double excitations were constructed within the π space applying the interacting space restriction.[1] No excitations from occupied σ orbitals and to virtual σ orbitals were allowed. This procedure was accomplished in the following way. Starting from a closed shell self consistent field (SCF) calculation, all occupied and virtual σ orbitals were frozen by transforming the one- and two-electron integrals into a new basis keeping only the π orbitals. The effect of the frozen σ orbitals was folded into effective one-electron Hamilton matrix elements according to the repartioning formalism of Shavitt.[2] The entire MCSCF and MR-AQCC steps were performed in this basis and the resulting natural orbitals were transformed back to the original atomic orbital basis. All calculations were performed in D_{2h} symmetry.

The occupation patterns of the CAS(8,8) used for the periacene and circumacene calculations were chosen such that for every irreducible representation (irrep) of the D_{2h} point group one strongly occupied and one weakly occupied orbital were present in the active space. Periacenes were arranged in the *xy*-plane where the armchair edge is extended along the *x*-axis and the zigzag edge along the *y*-axis. In this arrangement the π-orbitals have symmetries b_{1u}, b_{2g}, b_{3g} and a_u. In each of these irreps two orbitals were considered as active. For the (3a,*k*z) periacenes the number of doubly occupied π-orbitals amounted to (k, k, k - 1, k - 1) in the above order of irreps. For the (5a,*k*z) periacenes these amounted to $\left(\left\lfloor\frac{3k}{2}\right\rfloor + 1, \left\lceil\frac{3k}{2}\right\rceil, \left\lceil\frac{3k}{2}\right\rceil - 1, 3k2 - 1\right.$ where the symbols x and x are used to denote the largest integer number below x and the smallest integer number above x, respectively. Circumacenes were also arranged in the *xy*-plane where the armchair edge is extended along the *x*-axis and the zigzag edge along the y-axis. Again, one strongly and one weakly occupied orbital per irrep were included in the active space. For the (3a,*k*z) circumacenes the following number of orbitals per irrep were kept doubly occupied: (k + 1, k, k, k - 1). For the (5a,*k*z) circumacenes the number of reference doubly occupied (DOCC) orbitals amounted to $\left(\left\lfloor\frac{3k}{2}\right\rfloor + 2, \left\lceil\frac{3k}{2}\right\rceil + 1, \left\lceil\frac{3k}{2}\right\rceil, \left\lfloor\frac{3k}{2}\right\rfloor\right)$.

Polyacenes were arranged in the *xy*-plane with the long axis along the *x*-direction. The specific orbital occupations used in the RAS(6)/CAS(4,4)/AUX(6)-1ex scheme defining the reference wavefunction is given in Table S1. These occupations were chosen by considering the NO populations of initial MR-AQCC calculations based on a CAS(8,8) reference. It was observed that more than one weakly occupied NO of each of the b_{1u} and b_{2g} irreps was significantly occupied in these calculations and hence they were included in the active AUX space. The opposite trend, i.e. several strongly occupied NOs with a population of only about 1.9, was found for the b_{3g} and a_u irreps. Hence, these were included in the active RAS space. In the final RAS/CAS/AUX calculations it could, therefore, be assured that all orbitals with significant deviation from either zero or two were treated as active. For example even in the strongly polyradical 10-acene system all orbitals with occupations between 1.91 and 0.09 were contained in the RAS/CAS/AUX space.

The 6-31G basis set[3] has been used in all MR-AQCC calculations. Geometry optimizations were performed at the level of Møller-Plesset perturbation theory[4] using the resolution of the identity approach (RI-MP2)[5] and the SV(P) basis set.[6]

The MCSCF and MR-AQCC calculations were performed with a parallel version[7] of the COLUMBUS program system.[8] The RI-MP2 calculations have been performed with TURBOMOLE program.[9]

Table S1: Orbital occupation specification for the RAS(6)/CAS(4,4)/AUX(6)-1ex used in MCSCF and MR-AQCC calculations on polyacenes with k condensed rings.[a]

	b_{1u}	b_{2g}	b_{3g}	a_u
DOCC[b]	$\boldsymbol{\left\lfloor \frac{k}{2} \right\rfloor}$	$\boldsymbol{\left\lceil \frac{k}{2} \right\rceil - 1}$	$\boldsymbol{\left\lceil \frac{k}{2} \right\rceil - 3}$	$\boldsymbol{\left\lfloor \frac{k}{2} \right\rfloor - 3}$
RAS	**1**	**1**	**2**	**2**
CAS	1	1	**1**	**1**
AUX	2	2	1	1

[a] Strongly occupied orbitals are marked in bold.

[b] $\lfloor x \rfloor$ and $\lceil x \rceil$ are used to denote the largest integer number below x and the smallest integer number above x, respectively.

Table S2: D1 and D2 diagnostic values for the *n*-acene series using the CCSD method and the cc-pVDZ.[10] The threshold values are: D1 = 0.05 and D2 = 0.18.

Compound	D1	D2
2-acene	0.0309	0.203
4-acene	0.0360	0.224
6-acene	0.0399	0.240
8-acene	0.0423	0.253

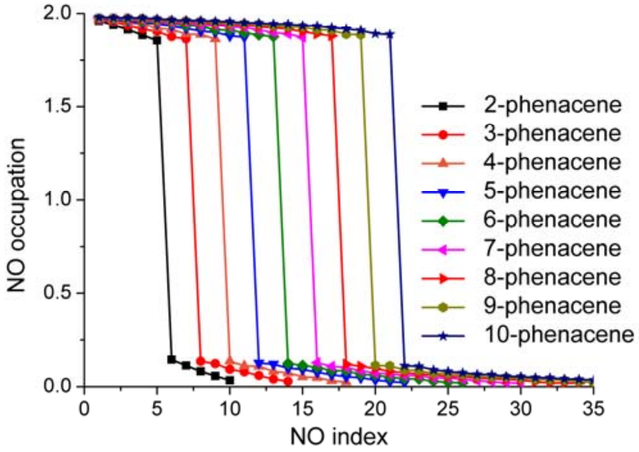

Figure S1. Evolution of MR-AQCC NO occupations with the length of the phenacene chain.

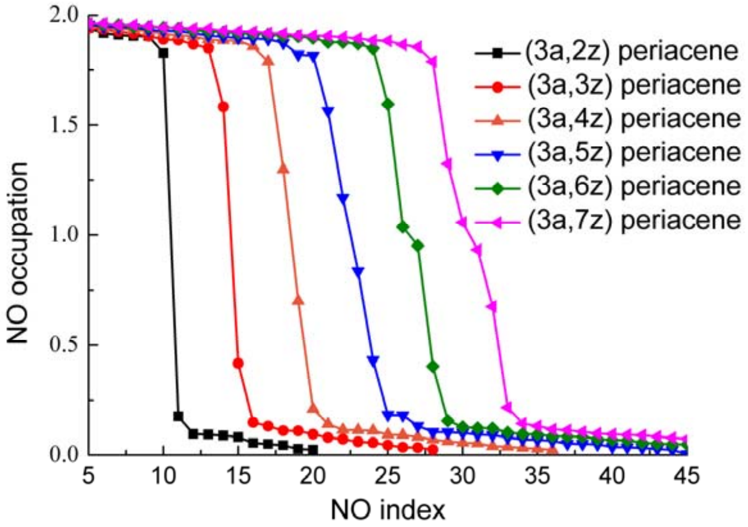

Figure S2. Dependence of MR-AQCC NO occupations on the zigzag length in (3a,*n*z) periacenes.

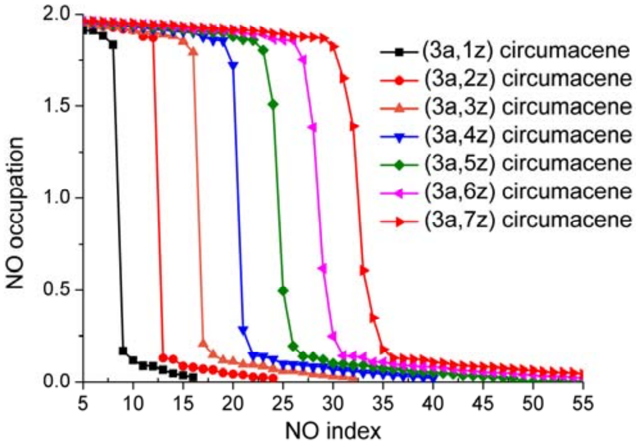

Figure S3. Dependence of NO occupations on zigzag length *n* in (3a,*n*z) circumacenes.

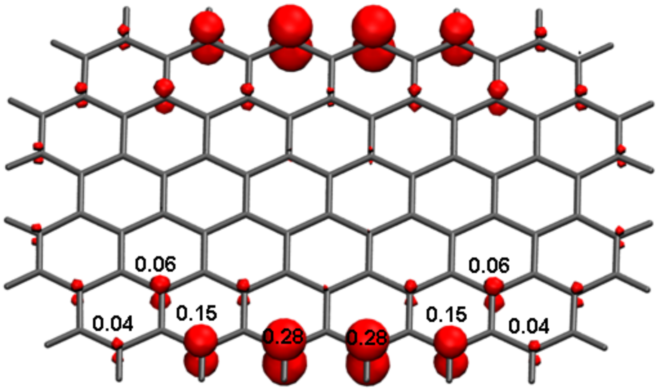

Figure S4. Unpaired electron density for (5a,6z) circumacene (isovalue 0.005 *e*). The total number of unpaired electrons N_U is 4.1 *e*, individual atomic values are given next to the respective carbon atoms.

References:

[1] A. Bunge, *J. Chem. Phys.* **1970**, *53*, 20-28.

[2] I. Shavitt, in *The Unitary Group for the Evaluation of Electronic Energy Matrix Elements, Lecture Notes in Chemistry, Vol. 22* (Ed.: J. Hinze), Springer, Berlin, **1981**, pp. 51-99.

[3] W. J. Hehre, R. Ditchfield, J. A. Pople, *J. Chem. Phys.* **1972**, *56*, 2257-2261.

[4] C. Moller, M. S. Plesset, *Phys. Rev.* **1934**, *46*, 0618-0622.

[5] a) O. Vahtras, J. Almlof, M. W. Feyereisen, *Chem. Phys. Lett.* **1993**, *213*, 514-518; b) F. Weigend, M. Haser, *Theor. Chem. Acc.* **1997**, *97*, 331-340.

[6] A. Schafer, H. Horn, R. Ahlrichs, *J. Chem. Phys.* **1992**, *97*, 2571-2577.

[7] a) H. Dachsel, H. Lischka, R. Shepard, J. Nieplocha, R. J. Harrison, *J. Comput. Chem.* **1997**, *18*, 430-448; b) T. Muller, *J. Phys. Chem. A* **2009**, *113*, 12729-12740.

[8] a) H. Lischka, R. Shepard, F. B. Brown, I. Shavitt, *International Journal of Quantum Chemistry* **1981**, *S15*, 91-100; b) H. Lischka, R. Shepard, R. M. Pitzer, I. Shavitt, M. Dallos, T. Müller, P. G. Szalay, M. Seth, G. S. Kedziora, S. Yabushita, Z. Y. Zhang, *Physical Chemistry Chemical Physics* **2001**, *3*, 664-673; c) H. Lischka, R. Shepard, I. Shavitt, R. M. Pitzer, M. Dallos, T. Müller, P. G. Szalay, F. B. Brown, R. Ahlrichs, H. J. Boehm, A. Chang, D. C. Comeau, R. Gdanitz, H. Dachsel, C. Ehrhardt, M. Ernzerhof, P. Höchtl, S. Irle, G. Kedziora, T. Kovar, V. Parasuk, M. J. M. Pepper, P. Scharf, H. Schiffer, M. Schindler, M. Schüler, M. Seth, E. A. Stahlberg, J.-G. Zhao, S. Yabushita, Z. Zhang, M. Barbatti, S. Matsika, M. Schuurmann, D. R. Yarkony, S. R. Brozell, E. V. Beck, J.-P. Blaudeau, M. Ruckenbauer, B. Sellner, F. Plasser, J. J. Szymczak, *COLUMBUS, an ab initio electronic structure program, release 7.0 (2012)*, www.univie.ac.at/columbus.

[9] a) TURBOMOLE V7.3 2012, a development of University of Karlsruhe and Forschungszentrum Karlsruhe GmbH, 1989-2007, TURBOMOLE GmbH, since 2007; available from http://www.turbomole.com; b) R. Ahlrichs, M. Bar, M. Haser, H. Horn, C. Kolmel, *Chem. Phys. Lett.* **1989**, *162*, 165-169.

[10] T. H. Dunning, *J. Chem. Phys.* **1989**, *90*, 1007-1023.
